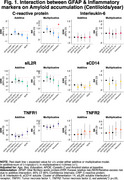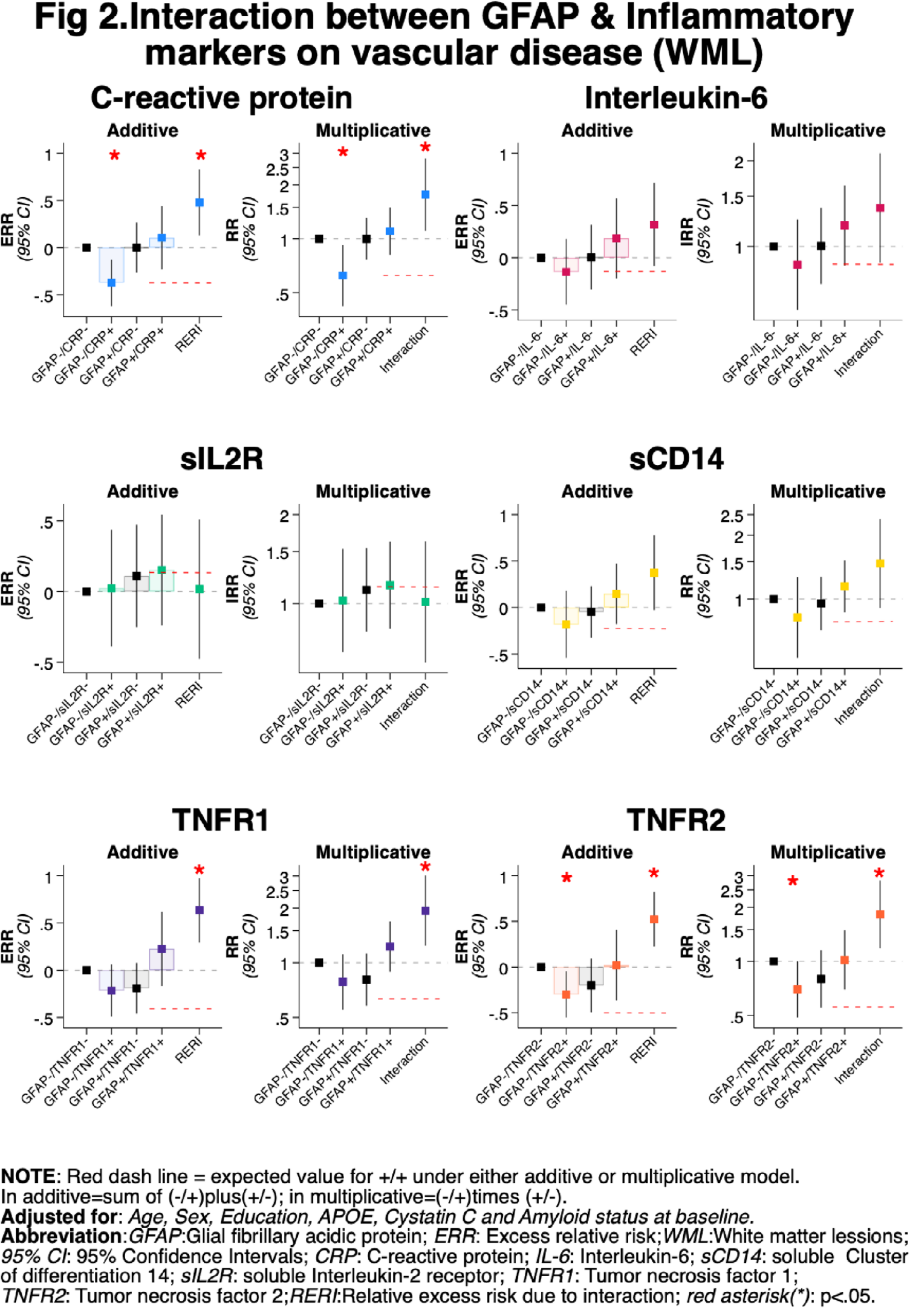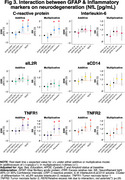# Synergistic effects of peripheral inflammation and neuroinflammation on amyloid, vascular, and neurodegeneration markers across the Alzheimer's disease spectrum

**DOI:** 10.1002/alz70856_099147

**Published:** 2025-12-24

**Authors:** Laura Alejandra Ramirez Tirado, Ann D Cohen, C. Elizabeth Shaaban, Cristy Matan, Brian J Lopresti, Milos D. Ikonomovic, Thomas K Karikari, Victor L. Villemagne, Oscar L Lopez

**Affiliations:** ^1^ University of Pittsburgh, Pittsburgh, PA, USA; ^2^ University of Pittsburgh Alzheimer's Disease Research Center, Pittsburgh, PA, USA; ^3^ University of Pittsburgh Alzheimer's Disease Research Center (ADRC), Pittsburgh, PA, USA; ^4^ University of Pittsburgh School of Medicine, Pittsburgh, PA, USA; ^5^ Department of Psychiatry, University of Pittsburgh, Pittsburgh, PA, USA; ^6^ VA Pittsburgh Healthcare System, Pittsburgh, PA, USA; ^7^ UPMC, Pittsburgh, PA, USA; ^8^ Institute of Neuroscience and Physiology, Department of Psychiatry and Neurochemistry, The Sahlgrenska Academy at University of Gothenburg, Mölndal, PA, Sweden; ^9^ The University of Pittsburgh, Pittsburgh, PA, USA

## Abstract

**Background:**

Systemic inflammation and astrogliosis are relevant in Alzheimer's disease (AD). We evaluated whether peripheral inflammation has synergistic effects with astrogliosis on β‐amyloid (Aβ), vascular, and neurodegeneration markers.

**Method:**

In the Ginkgo Evaluation of Memory Study, we evaluated the relationship between peripheral inflammation (TNF‐R1, TNF‐R2, IL‐6, IL‐2, CRP and sCD14) and astrogliosis (GFAP </≥196pg/mL) as to Aβ burden (</≥20centiloids) and accumulation (centiloids/year), white matter lesion (WML) volume, and neurodegeneration (NfL) across the AD spectrum. Participants underwent PiB‐PET scans between 2009‐2018. GFAP, NfL, and peripheral inflammatory markers were measured in 2009 by immunoassay; their values were standardized within the cohort. We used negative binomial regression models and adjusted for age, sex, education, *APOEε4*, cystatin C and baseline Aβ status.

**Result:**

190 participants were included (mean age: 86±2.8 yrs., 40.8% women, 96.9% White). GFAP was higher among Aβ+ participants; it did not differ by levels of peripheral inflammation. Aβ accumulation was slower among those with high sCD14, particularly among Aβ+. There was a significant negative multiplicative interaction between GFAP and CRP on Aβ accumulation (RR;95%CI:0.61;.38‐.99;p=0.047). WML were larger among GFAP+ individuals. There were significant additive and multiplicative interactions of GFAP with CRP (relative excess risk due to interaction (RERI);95%CI:0.47;0.12‐0.83;p=0.008; RR;95%CI:1.76;1.11‐2.81;p=0.016, respectively), with TNFR1 (RERI;95%CI:0.63;0.29‐0.97;p<.001; RR;95%CI:1.93;1.24‐3.01;p=0.004, respectively), and with TNFR2 (RERI;95%CI:0.52;0.22‐0.82;p=0.001; RR;95%CI:1.82;1.18‐2.82;p=0.006, respectively) on WML. Regarding neurodegeneration, there were significant joint effects of GFAP with CRP, sCD14 and/or TNFR1; and significant additive and/or multiplicative interactions of GFAP with CRP (RERI;95%CI:0.35;0.07‐0.62;p=0.012, RR;95%CI:1.37;1.04‐1.81;p=0.023, respectively) with TNFR1 (RERI;95%CI:0.29;0.08‐0.50;p=.007, RR;95%CI:1.30;1.03‐1.63;p=0.023, respectively) and with TNFR2 (RERI;95%CI:0.22;0.005‐0.45;p=.045).

**Conclusion:**

In this 85+ population, with expected high levels of inflammation and more co‐pathology; peripheral inflammation (CRP, TNFR1 and/or TNFR2) showed additive/multiplicative synergistic effects with astrogliosis resulting in less amyloid accumulation, greater vascular burden, and more neurodegeneration, particularly in Aβ+ participants. More inflammation may relate with more pathogenesis, bringing participants closer to the amyloid asymptote, slowing Aβ accumulation.